# The mediating and moderating effects of resilience on the relationship between sleep quality and psychological distress in Chinese women with infertility

**DOI:** 10.1186/s12905-024-03018-x

**Published:** 2024-03-21

**Authors:** Zhenhua Jiang, Sen Hou, Yajie Zhang, Liping Zong

**Affiliations:** 1 The Affiliated Hospital of Shandong University of Traditional Chinese Medicine, Jingshi Road, Jinan, 250014 Shandong China; 2 Jinan Maternity and Child Care Hospital, Jingsan Road, Jinan, 250000 Shandong China

**Keywords:** Infertility, Sleep quality, Resilience, Psychological distress

## Abstract

**Background:**

Research has widely indicated that the psychological distress experienced by infertile patients during fertility treatments may have a negative effect on the results of assisted reproduction. Although numerous studies have shown that psychological resilience and sleep quality are important influencing factors for psychological distress, the mediating mechanisms of psychological resilience in the relationship between sleep quality and psychological distress for Chinese women in particular remain unclear. Therefore, the current study investigates the association between sleep quality, resilience, and psychological distress in Chinese women with infertility and examines the mediating and moderating roles of resilience on the relationship between sleep quality and psychological distress.

**Methods:**

In this cross-sectional study, a total of 595 women with infertility who were undergoing IVF-ET were recruited at the Reproductive Medicine, Shandong University, from April to November 2019. Participants were instructed to complete four questionnaires, including a questionnaire about socio-demographic and clinical-related information, the Pittsburgh Sleep Quality Index (PSQI), the 10-item Connor-Davidson Resilience Scale (CD-RISC-10), and the Kessler-10 (K10). Pearson’s correlation analysis was conducted preliminarily to describe the relationships between sleep quality, resilience, and psychological distress. A mediation model and a moderated model were constructed and analyzed using the PROCESS macro for SPSS. The Johnson-Neyman (J-N) technique was then used to identify the regions of significance across the levels of moderator values.

**Results:**

Patients in the sample had a high prevalence of psychological distress (48.6%, K10 scores > 22), and mediation analysis indicated that resilience played a partially mediating role in the relationship between sleep quality and psychological distress (indict effect = 0.072, *P* < 0.001). Moderation analysis indicated that resilience also moderated the association between sleep quality and psychological distress.

**Conclusions:**

Resilience may play a key role in the relationship between sleep quality and psychological distress. Our findings imply that resilience training may therefore be an effective component of psychological distress intervention in women with infertility.

## Introduction

Infertility is a condition characterized by the failure to establish a clinical pregnancy after 12 months of regular and unprotected sexual intercourse or the impairment of a person’s capacity to reproduce either as an individual or with his/her partner [1]. According to the ‘China Infertility Investigation Report’, one in eight couples was diagnosed with infertility, and there were more than 40 million infertility patients in China [2]. Numerous studies have shown that infertile couples, and females in particular frequently experience psychological stress due to infertility and its treatment. This stress may interfere with adjustment processes and cause psychological distress, which is a state of emotional suffering characterized by depression and anxiety [3, 4]. Assisted reproductive technologies (ART) give hope to infertile couples and have become a realistic option for those seeking help to conceive. In vitro fertilization embryo transfer (IVF-ET) is one of the main therapies used to treat infertility, although it is complex and invasive, especially when procedures such as ovarian stimulation, oocyte retrieval, embryo transfer, and pregnancy tests after embryo transfer are performed [5]. In clinical work, researchers have found that patients often face great mental stress prior to oocyte retrieval, including concerns about the number of oocytes retrieved and embryo quantity and quality, which can lead to psychological distress. Importantly, there is also growing evidence that such psychological distress may have a negative effect on the results of IVF-ET treatment. It not only can reduce the pregnancy and live birth rates [3], but can also increase the risk of long-term negative emotions [6]. Therefore, greater attention to psychological distress and its related factors may be needed for women with infertility prior to oocyte retrieval.

Sleep disturbances can include short sleep duration, sleep continuity disturbance, circadian dysrhythmia, and poor sleep quality. About 20% of women have experienced sleep disturbances at some point from age 20 to age 40 [7], and this percentage increases with age [8]. One previous study showed that both objective and subjective sleep were associated with negative emotions [9]. Similarly, Rezaei et al. [10] showed that poor sleep quality is common among medical students and that it adversely affects their mental health. In addition, a longitudinal study from the Penn State Adult Cohort revealed a significant relationship between insomnia and incidents of depression using objective sleep measures [11]. Interestingly, sleep disturbances are thought to be frequent in women undergoing infertility treatment [12]. Recent studies in women undergoing in vitro fertilization-embryo transfer (IVF-ET) have found that 23% and 46% of women had poor sleep during oocyte pickup and ET, respectively [13]. For women specifically, sleep disorders have been found to be correlated with deteriorated reproductive capacity, increased menstrual dysfunction, spontaneous abortion, and adverse birth outcomes [14, 15]. However, research on the relationship between sleep quality and psychological health in women with infertility is limited.

Resilience is defined as an individual’s ability to maintain or restore relatively stable psychological and physical functioning when confronted with stressful life events and adversity [16], and previous studies have reported that resilience is negatively related to both sleep disorders [17] and psychological distress (e.g., anxiety and depression) [18]. For example, the A-CHILD study revealed that children with irregular bedtimes on weekdays demonstrated a low level of resilience [19]. A prospective, longitudinal study also found that the higher the rhythmicity of sleep in early childhood, the better the resilience outcomes in young adulthood [20]. Furthermore, recent research has shown that resilience is conducive to psychological health [21, 22]. For instance, a cross-sectional survey of 533 Ghanaian adolescents showed that enhancing resilience may improve the symptoms of anxiety and depression in the general adolescent population [21]. Similarly, Ding et al. [23] showed that resilience buffers the effect of depressive symptoms in Chinese children, and Kishore et al. [24] reported that resilience has a protective effect against depression during pregnancy. These findings suggest that resilience, as a defense mechanism, protects against negative emotions. Given the findings of these previous studies, we speculate that resilience may also be a protective factor in the relationship between poor sleep and psychological distress. However, no published study has yet tested how resilience interacts with sleep quality and psychological distress in women with infertility.

The present study therefore aims to evaluate the role of resilience in the relationship between sleep quality and psychological distress in Chinese women with infertility. In accordance with the aforementioned literature, we formulated the following hypotheses: (a) sleep quality is associated with psychological distress, and resilience (b) mediates and also (c) moderates the relationship between sleep quality and psychological distress (Fig. 1). We expect that if true these hypotheses will provide new insights into improving mental health in women with infertility.

**Fig. 1A:**
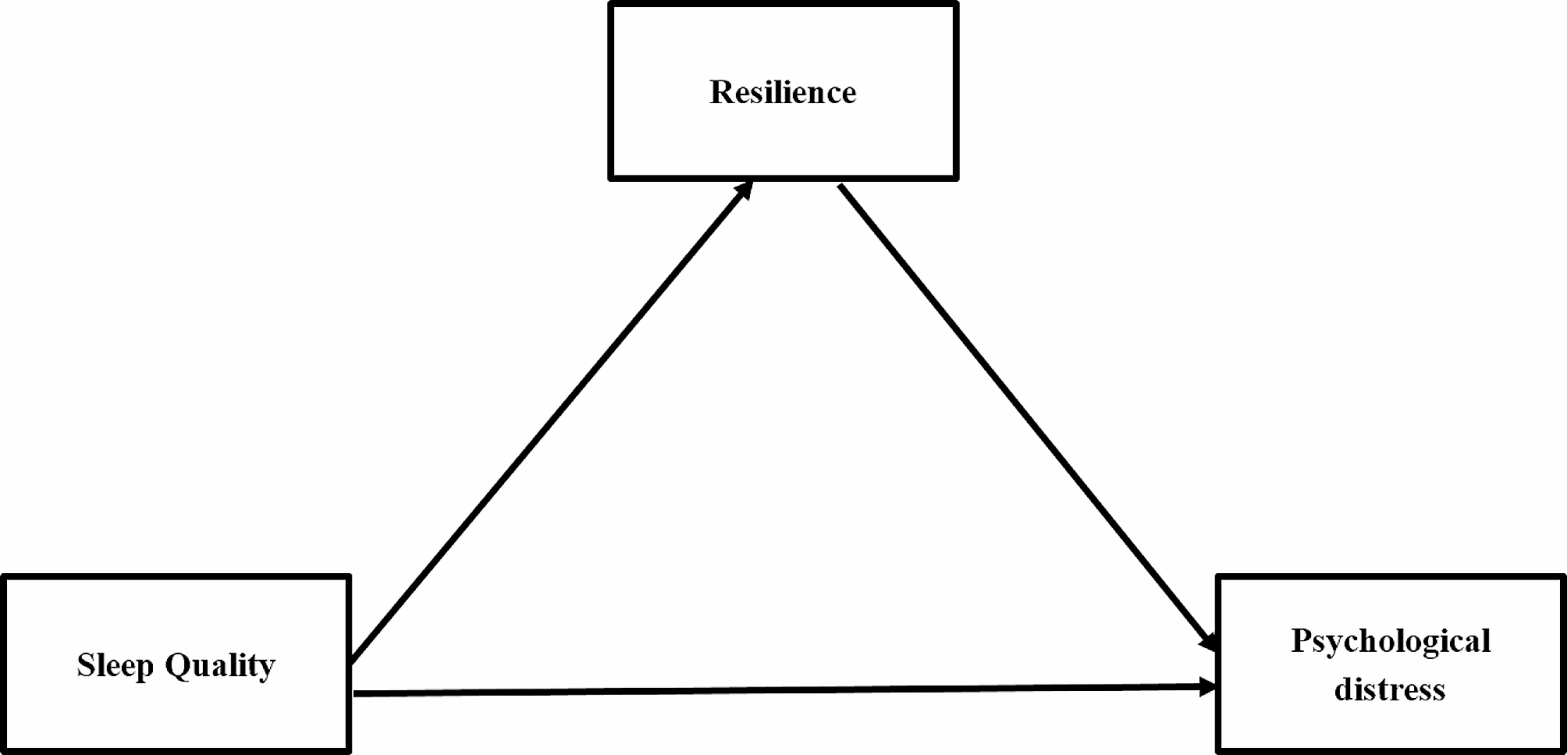
The proposed theoretical diagram of mediation model in the study

**Fig. 1B:**
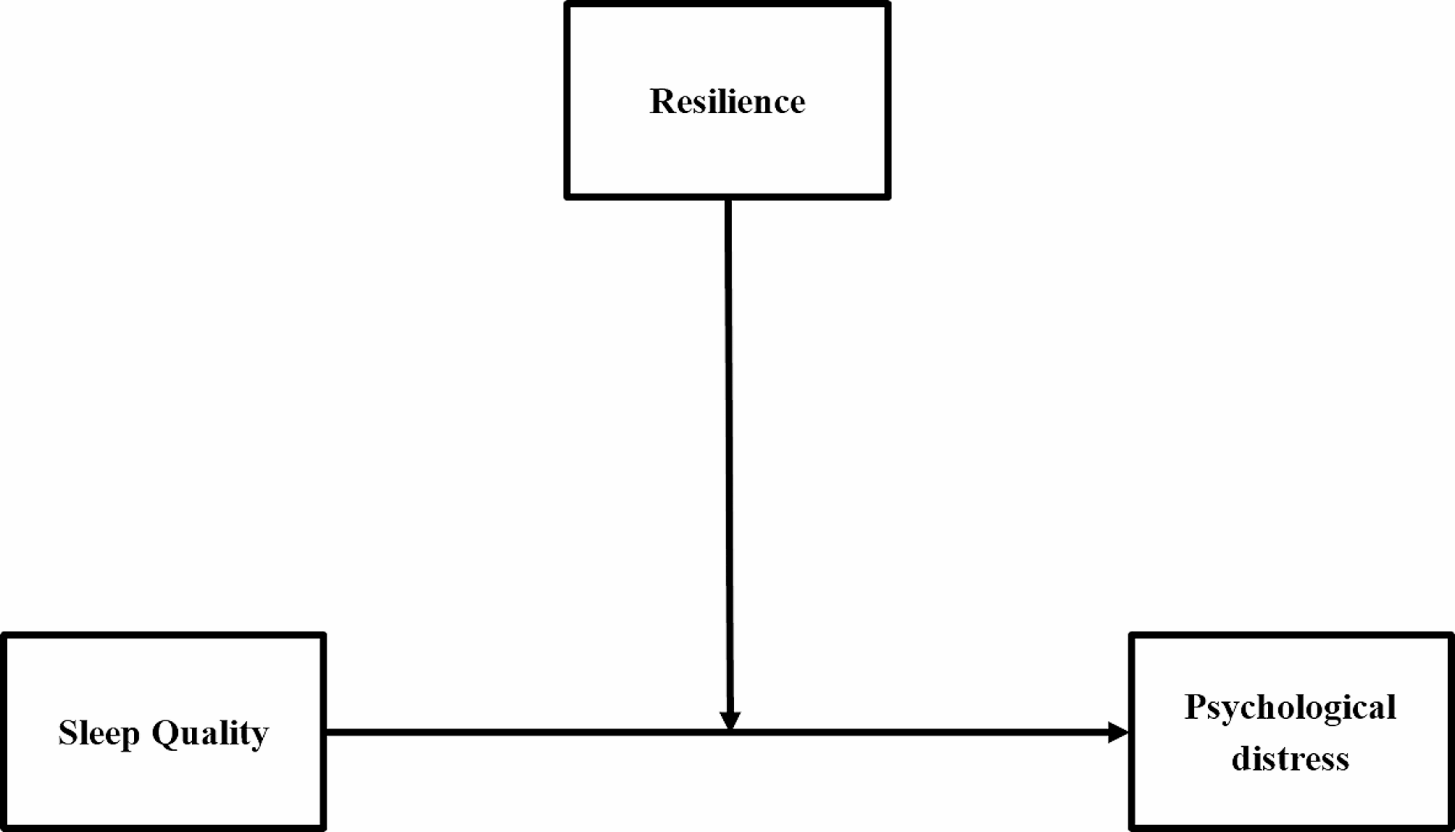
The proposed theoretical diagram of moderation model in the study

## Methods

### Design and data collection

This cross-sectional study was conducted in the infertility outpatient departments in the Center for Reproductive Medicine, Shandong University, among patients who underwent IVF-ET from April to November 2019. The inclusion criteria were age ≥ 20 years, diagnosis of infertility and first cycle of ART treatment, ability to understand and answer the questionnaires, and willingness to participate. This study was approved by the ethics committees of the Reproductive Medical Center of Shandong University.

Prior to oocyte retrieval, women with infertility who were to undergo HCG injections (this is required to promote further maturation of the oocyte) were consecutively recruited by nurses. Our researchers, fully trained and certified, recruited participants according to the inclusion criteria and then explained the purpose of the study and investigation procedure in Chinese. We used the Chinese versions of the following questionnaires to conduct a face-to-face survey of participants. Researchers checked to see if the questionnaires were completely and properly filled out. A total of 620 women were invited to participate in this investigation. Among them, 15 provided incomplete surveys, and 10 refused participation, so 595 were included in the final analysis. Participants’ information included age, education, occupation, children adopted, and infertility duration and diagnosis.

### Measures

#### Sleep quality

The Pittsburgh Sleep Quality Index (PSQI) was used to assess individuals’ subjective sleep quality during the past month [25]. It consists of a 19-item self-report questionnaire and includes seven components: subjective sleep quality, sleep latency, sleep duration, habitual sleep efficiency, sleep disturbances, use of sleeping medications, and daytime dysfunction. The total score of the PSQI ranges from 0 to 21, with higher scores indicating lower levels of sleep quality. The PSQI has demonstrated good reliability with a Crobachs alpha of 0.85 [26]. For the total scale, Cronbach’s alpha was 0.72 in this study.

### Resilience

The 10-item Connor-Davidson Resilience Scale (CD-RISC-10) was used to evaluate resilience [27, 28]. Ten items in total are rated on a 5-point Likert scale, ranging from 0 (never) to 4 (always), with higher scores reflecting higher levels of resilience. The Chinese version showed good reliability and validity [27]. Cronbach’s alpha was computed to be 0.87 in this study.

### Psychological distress

The original Kessler-10 rating scale (K10) comprises 10 items rated on a five-point Likert scale from 1 (hardly) to 5 (very much) [29]. K10 is widely used to measure individuals’ psychological distress, with higher scores reflecting higher negative emotions. In this study, a total score greater than 22 indicates psychological distress [30]. This scale was reliable and specific for screening serious mental illnesses.The Chinese version of the K10 is regarded as a good measurement of psychological distress with good reliability and validity [31]. Cronbach’s alpha was 0.81 in the present study.

### Statistical analysis

SPSS (version 22.0) and PROCESS (version 3.3) were used to analyze the data, and the sample characteristics were assessed with descriptive statistics. Moreover, independent *t*-tests and one-way ANOVA were performed to compare the differences in the psychological distress levels by characteristic variables. Pearson’s correlation analyses were also conducted to preliminarily describe the associations among sleep quality, resilience, and psychological distress. Following Hayes’ guidelines, the SPSS PROCESS macro was used to test the mediating and moderating effects of resilience on the relationship between sleep quality and psychological distress. The significance of the indirect effect was tested using bootstrapping confidence intervals (CIs). If the 95% bias-corrected CI from 5,000 bootstrap samples did not contain zero, then the indirect effect was significant. On the contrary, the interaction effect is automatically calculated through the PROCESS macro. The Johnson-Neyman (J-N) technique was also used to further identify the regions of significance across the level of the moderator value. In this study, *P* values < 0.05 (two-tailed) were regarded as statistically significant.

## Results

### Participants’ demographic characteristics and prevalence of psychological distress

Table 1 shows the sample demographic information and differences in the global K10 scores. The mean age of the women was 31.66 years (SD = 4.63, range 20 ∼ 48). For education, 57.6% of the participants had finished high school, and fewer than half had a college or higher degree. For occupation, 46.9% of the respondents were unemployed. The K10 scores were different depending on whether the child had child or not (t = 2.041, *P* = 0.037). Pearson’s correlation analysis showed that the K10 scores were not significantly different from other demographic information. The descriptive results for the PSQI, resilience, and K10 variables are depicted in Table 2. The mean score of the K10 was 21.29 (SD = 6.11), and 289 participants (48.6%) reported psychological distress (K10 scores > 22). Furthermore, the overall score of the PSQI was 4.72 (SD = 2.43), and the mean score of resilience was 21.29, indicating its moderate levels.

**Table 1:** Characteristics of the participants and the comparisons of the scores on K10

Variables	N(%)	K10 M (SD)	t/F	P
Age			0.913	0.304
<35years	442(74.3%)	21.49 ± 5.93		
35-42years	126(21.2%)	20.38 ± 6.08		
≥ 43years	27(4.5%)	21.84 ± 6.23		
Education			1.565	0.210
Less than high school	343(57.6%)	21.93 ± 6.19		
College graduate or higher	252(42.4%)	20.91 ± 6.03		
Occupation			-1.495	0.135
Employee	307(53.1%)	20.93 ± 5.87		
Unemployed	288(46.9%)	21.67 ± 6.34		
Have child or not			2.041	0.037*
Yes	164(27.6%)	20.80 ± 6.55		
No	431(72.4%)	21.76 ± 6.04		
Duration of infertility			0.268	0.789
< 3years	242(40.7%)	21.37 ± 6.01		
≥ 3years	353(59.3%)	21.24 ± 6.18		
Infertility diagnosis			0.860	0.462
Female factor	430(72.3%)	21.51 ± 6.11		
Male factor	82(13.8%)	20.38 ± 6.04		
Couple factor	47(7.9%)	21.19 ± 6.07		
Unexplained	36(6.1%)	20.86 ± 6.45		

*P < 0.05

**Table 2:** Correlations of sleep quality, Resilience, and psychological distress

Variables	M ± SD	PSQI	Resilience	K10
PSQI	4.72 ± 2.43	1	-	-
Resilience	36.62 ± 7.08	-0.192^**^	1	-
K10	21.29 ± 6.11	0.340^**^	-0.425^**^	1

**P < 0.01

### Correlations among sleep quality, resilience, and psychological distress

Pearson’s correlation coefficients among this study variables are presented in Table 2. PSQI scores were negatively associated with resilience (*r* = -0.192, *P* < 0.001) and were positively associated with K10 scores (*r* = 0.340, *P* < 0.01), and resilience scores were negatively correlated with K10 scores (*r* = -0.425, *P* < 0.001).

### Testing for the mediating effect of resilience

**Fig. 2:**
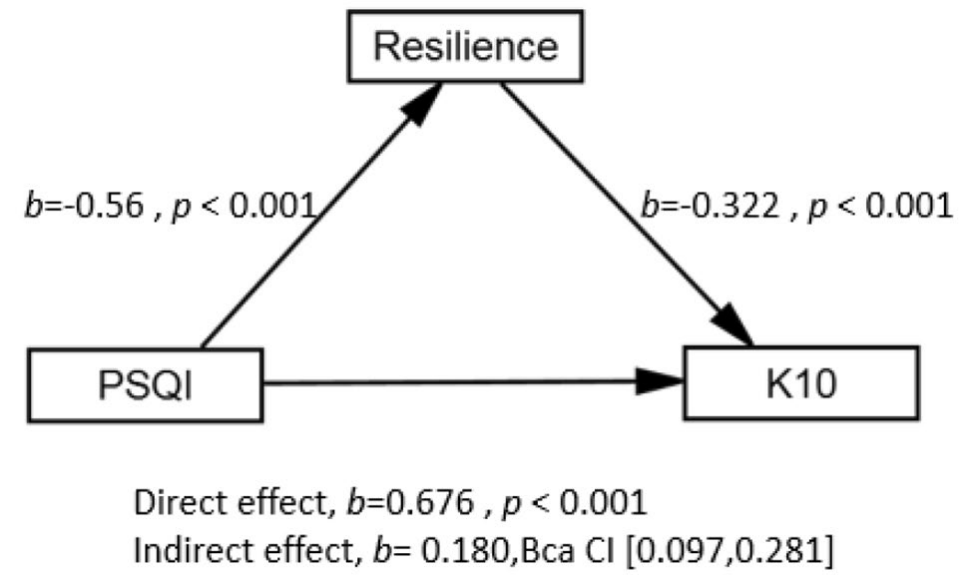
Diagram of the Mediation Model with Regression Coefficients, Indirect Effect, and Bootstrapped CIs.

A statistical mediation analysis was conducted to determine if resilience mediates the relationship between sleep quality and psychological distress. As shown in Fig. 2, resilience did statistically mediate such a relationship because the bootstrap samples did not include zero. The direct effect of resilience on the K10 scores was still significant (B = 0.269, t = 7.411, *P* < 0.01). On the contrary, the standardized indirect effect value of the PSQI scores on the K10 through resilience was 0.072, accounting for 21.17% of the total effect value (0.340).

### Testing for the moderating effect of resilience

**Fig. 3:**
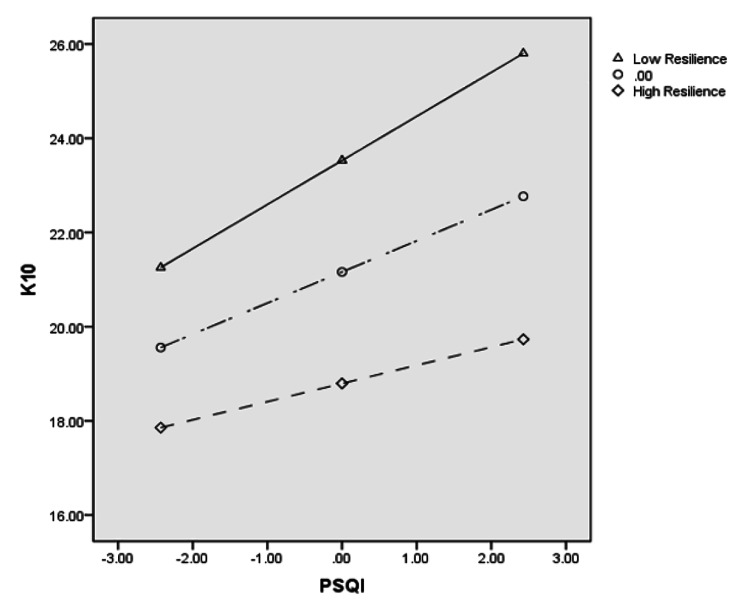
Resilience moderates the effect of PSQI on K10

**Fig. 4:**
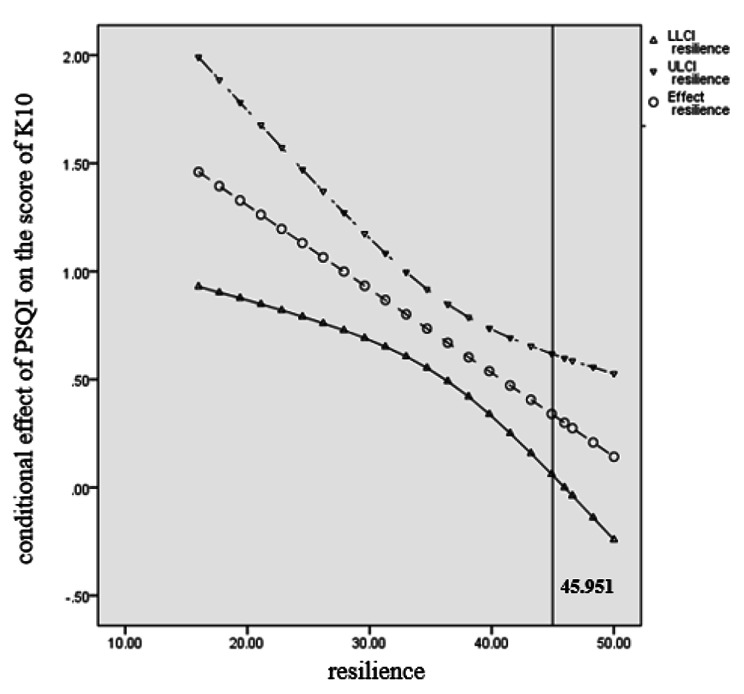
Visual representation of the conditional effect of PSQI on K10 as moderated by resilience

A multiple regression analysis was conducted to determine if resilience moderates the relationship between sleep quality and psychological distress. Consequently, a statistically significant interaction was found: F = 69.989, *P* < 0.001, R squared = 0.262 (Table 3). As depicted in Fig. 3, when resilience was low, the association of PSQI and K10 was strong (*b* = 0.935, 95% CI [0.692, 1.178], *t* = 7.560, *P* < 0.001). When resilience was at its mean, the association between PSQI and K10 was moderate (*b* = 0.661, 95% CI [0.483, 0.839], *t* = 7.284, *P* < 0.001). Finally, when resilience was high, the association between PSQI and K10 was mild (*b* = 0.386, 95% CI [0.129, 0.643], *t* = 2.953, *P* = 0.003). The Johnson-Neyman technique further indicated that the conditional effect of the PSQI on the K10 was statistically significant for women with infertility scoring lower than 45.951 on resilience (Fig. 4).

**Table 3:** Multiple Regression Analysis Summary for PSQI and K10 with Moderated by Resilience

DV	IV	Coeff	SE	t	P	LLCI	ULCI
K10	constant	21.163	0.220	96.328	0.000	20.731	21.594
	Resilience	-0.334	0.031	-10.677	0.000	-0.395	-0.273
	PSQI	0.661	0.091	7.284	0.000	0.483	0.839
	Resilience*PSQI	-0.039	0.013	-3.077	0.002	-0.064	-0.014

Note: R^2^ = 0.262; F = 69.989, *P* < 0.001; DV: dependent variable; IV: independent variable

## Discussion

This study explored the psychological distress of women with infertility during their ART treatment period and the relationships between sleep quality, resilience, and psychological distress. The results showed that 48.6% of the participants reported psychological distress, which was higher compared to that in previous studies [32, 33]. Chiaffarino [30] found that 17.9% of women undergoing ART in Italy had depressive symptoms and 14.7% had anxious symptoms. Moreover, Chen [33] reported that 40.2% of 112 women with infertility had a psychiatric disorder. Although cultural differences between various geological locations exist, these findings suggest that psychological distress is common. In addition, the negative emotions of infertile patients are associated with an increased risk of adverse IVF outcomes and long-term mental health issues [34]. Thus, the psychological distress in women with infertility is worthy of concern at the start of treatment, and psychosocial care should be delivered during treatment in order to improve patients positive psychological adjustment.

We also found that sleep quality was associated with psychological distress. Specifically, sleep is essential for maintaining mental health, and our findings are similar to those of many previous studies. For instance, a 9-year national representative longitudinal study showed that there was a significant increased risk of new-onset depression and anxiety in women who reported frequent sleeping difficulties [35]. Moreover, a cross-sectional study also reported a strong degree of correlation between poor sleep quality and negative emotions, such as depression and anxiety, among women undergoing reproductive treatments [36]. In addition, another study found a bidirectional relationship between insomnia and psychological distress, which shows both that anxiety and depression are related to future insomnia and that insomnia may be a risk factor for future anxiety and depression [37]. One possible explanation for this relationship is that sleep disturbances may alter cognition, resulting in negative emotions. From a biological perspective, sleep is influenced by circadian rhythms, which may contribute to the decrease in diurnal mood in circadian dysregulation [35]. Crucially, although sleep disorders and psychological distress are related to the treatment outcomes of women with infertility [14, 15, 38, 39], sleep quality may be an adjustable factor. Therefore, every effort should be made to improve sleep quality during fertility treatment.

Our findings also indicate that sleep quality has both direct and indirect effects on psychological distress through resilience. Resilience thus plays a mediating role between sleep quality and psychological distress. Among women with infertility, poor sleep was associated with low levels of resilience, which were correlated with high levels of psychological distress. The potential mechanism for this may be that lack of sleep can activate the hypothalamic-pituitary-adrenal cortex axis and renin-angiotensin system, which are associated with increasing the cortisol and angiotensin II awakening responses, thereby causing anxiety and depressive neurosis [40, 41]. However, resilience may effectively alleviate this neurohormonal response [41]. Given the precedent that positive psychology has been successfully used in the prevention and intervention of psychological distress, the latent mediating mechanism of resilience on sleep quality and psychological distress may provide new insights into improving mental health in women with infertility.

Furthermore, our moderation analysis demonstrated the moderating role of resilience between sleep quality and psychological distress. Our results indicated that resilience may buffer the effect of sleep disturbance on psychological distress, which is inconsistent with a previous study [42]. Esi van der Zwan et al. reported that resilience did not affect any of the relationships between sleep duration and anxiety during pregnancy. One of the reasons for this difference may be that the prevalence of sleep disturbances greatly depends on the method of measuring sleep quality. Our study revealed that a high level of resilience was protective against an increase in anxiety/depression when sleep quality deteriorated. Resilient individuals were adept at drawing on positive emotions in unfavorable circumstances, which may have buffered the effect of negative psychological distress and helped them to maintain better well-being outcomes [43, 44]. Interestingly, one feature of resilience is that it can be enhanced through purposeful training [45]. Thus, our results suggest that resilience training may be an effective type of intervention [46] for improving sleep disorders in women with infertility.

### Strengths and limitations

This is the first study to our knowledge to examine the relationships between sleep quality and psychological distress among Chinese women with infertility. Prior research has revealed that sleep quality and resilience are significant factors that influence psychological distress in patients with infertility, and this implies that healthcare professionals, including medical and nursing staff, should not only focus on treatment plans and pregnancy outcomes but also prioritize the sleep quality of patients during the infertility treatment process. Mindfulness relaxation training can also be employed to assist patients in alleviating stress throughout their journey towards conception [47]. Furthermore, studies on psychological resilience have emphasized the importance of both internal and external protective factors [48, 49]. Internal protective factors encompass self-esteem, a positive temperament, individual self-efficacy, and self-confidence. Therefore, health education and psychological counseling can be utilized to provide necessary support to patients by guiding them in managing their emotions effectively while enhancing their confidence when dealing with infertility. External protective factors encompass family and social support. Nurses should thus actively encourage patients to confide their concerns in their family members, particularly their spouses. Family resilience may help to buffer the impact of fertility-related stress on psychological distress in IVF-ET patients [50] and thus warrant further exploration of related interventions. Infertility treatment is a protracted process that might involve multiple oocyte retrieval and embryo transfer cycles for many patients. Therefore, attending to the psychological distress experienced by first IVF-ET treatment recipients and proposing effective targeted strategies can facilitate better adaptation to the long-term treatment journey many women face.This study has several limitations to be considered. First, due to the nature of its cross-sectional design, the causal relationships between sleep quality, resilience, and psychological distress could not be ascertained. Thus, in the future, longitudinal or experimental designs should be employed. Second, the type of measurement used in the present study was self-reporting, which can result in common method variance. Consequently, objective measures for assessing sleep quality, such as polysomnography [51], should be adopted in future studies. Third, because our participants were undergoing their first cycle of ART, the results may not be stable across multi-cycle treatment. Fourth, this study was a single-center survey, and the results need to be investigated using multi-center and large-sample studies in order to determine their generalizability. Finally, future studies should also utilize infertility-specific scales to mitigate potential reporting bias.

## Conclusion

In this study, we found that Chinese females preparing for their first cycle of ART treatment were at risk of psychological distress, especially those who had no children. The study provides researchers with an empirical framework with which to improve patient psychological distress by discussing the mediating and moderating effects of resilience between sleep quality and psychological distress. As a result, future interventions that seek to improve psychological distress among women with infertility should include improving sleep quality and increasing resilience as primary intervention targets.

## Data Availability

The data that supports the findings in this study is available from the corresponding author upon reasonable request.

## Abbreviations

ART: Assisted Reproductive Technologies
PSQI: Pittsburgh Sleep Quality Index
IVF-ET: In Vitro Fertilization-Embryo Transfer
CD-RISC: Connor-Davidson Resilience Scale
